# Alitretinoin – molecular and cellular mechanisms of action

**DOI:** 10.1186/1479-5876-9-S2-P16

**Published:** 2011-11-23

**Authors:** Andreas Kislat, Stephan Meller, Rodrigo Mota, Peter A Gerber, Bettina A Buhren, Erich Bünemann, Ulrike Wiesner, Thomas Ruzicka, Bernhard Homey

**Affiliations:** 1 Dept. of Dermatology, Heinrich-Heine University Hospital, Duesseldorf, Germany; 2 Dept. of Dermatology, Ludwig-Maximilians-University, Munich, Germany

## Background

Chronic hand eczema (CHE) represents an inflammatory skin disease with a high prevalence ranging from 7-12% in Western industrialized countries. It was only starting from 2008 that the first, and until now the only, systemic treatment option, i.e. oral alitretinoin was approved in several countries for severe CHE unresponsive to potent topical corticosteroids. However, as the precise mechanism of actions (MOA) of alitretinoin in CHE are so far unknown, we undertook following investigations in order to shed some light on potential underlying immunomodulatory mechanisms.

## Materials and methods

To investigate the mode of action of alitretinoin we stimulated human primary keratinocytes as well as leukocyte subsets and performed quantitative real-time PCR analyses, FACS-analyses, cell-proliferation assays as well as mixed leukocyte reactions (MLR).

## Results

Alitretinoin acts on keratinocytes as well as dendritic cells. Keratinocytes show a significant reduction of chemokine expression after stimulation with alitretinoin whereas on dendritic cells alitretinoin inhibits the upregulation of the maturation marker CD83 as well as the co-stimulatory molecules CD80 and CD86. Consequently, alitretinoin-treated dendritic cells show significantly impaired T cell activating properties. Further analyses demonstrated that the immunomodulatory effect of alitretinoin results in a marked suppression of allogenic proliferation of leukocytes in MLR.

## Conclusions

Taken together, these results suggest that alitretinoin modulates innate as well as adaptive immune responses by suppression of chemokine-induced leukocyte recruitment and inhibition of dendritic cell-mediated T cell activation.

